# Regional analysis to delineate intrasample heterogeneity with RegionalST

**DOI:** 10.1093/bioinformatics/btae186

**Published:** 2024-04-05

**Authors:** Yue Lyu, Chong Wu, Wei Sun, Ziyi Li

**Affiliations:** Department of Biostatistics, The University of Texas MD Anderson Cancer Center, Houston, TX 77030, United States; Department of Biostatistics and Data Science, The University of Texas Health Science Center at Houston, Houston, TX 77030, United States; Department of Biostatistics, The University of Texas MD Anderson Cancer Center, Houston, TX 77030, United States; Biostatistics Program, Public Health Science Division, Fred Hutchinson Cancer Center, Seattle, WA 98109, United States; Department of Biostatistics, University of North Carolina at Chapel Hill, Chapel Hill, NC 27516, United States; Department of Biostatistics, University of Washington, Seattle, WA 98195, United States; Department of Biostatistics, The University of Texas MD Anderson Cancer Center, Houston, TX 77030, United States

## Abstract

**Motivation:**

Spatial transcriptomics has greatly contributed to our understanding of spatial and intra-sample heterogeneity, which could be crucial for deciphering the molecular basis of human diseases. Intra-tumor heterogeneity, e.g. may be associated with cancer treatment responses. However, the lack of computational tools for exploiting cross-regional information and the limited spatial resolution of current technologies present major obstacles to elucidating tissue heterogeneity.

**Results:**

To address these challenges, we introduce RegionalST, an efficient computational method that enables users to quantify cell type mixture and interactions, identify sub-regions of interest, and perform cross-region cell type-specific differential analysis for the first time. Our simulations and real data applications demonstrate that RegionalST is an efficient tool for visualizing and analyzing diverse spatial transcriptomics data, thereby enabling accurate and flexible exploration of tissue heterogeneity. Overall, RegionalST provides a one-stop destination for researchers seeking to delve deeper into the intricacies of spatial transcriptomics data.

**Availability and implementation:**

The implementation of our method is available as an open-source R/Bioconductor package with a user-friendly manual available at https://bioconductor.org/packages/release/bioc/html/RegionalST.html.

## 1 Introduction

Cancer treatment failure is frequently linked to the molecular heterogeneity of tumors, characterized by diverse mutation profiles, gene expression levels, and biological features (Kim and Tannock 2005, Prasetyanti and Medema 2017). Spatial transcriptomics, which allows gene expression quantification while preserving spatial location information, has emerged as a powerful tool for understanding the expression of genes within their native tissue context (Burgess 2019). Spatial transcriptomics has shown promise in elucidating the molecular mechanisms underlying tumor heterogeneity and could potentially help overcome treatment failure in cancer (Berglund *et al.* 2018, Zhao *et al.* 2021).

A fundamental problem in spatial transcriptomics is understanding cell heterogeneity in a sample slice (Rao *et al.* 2021). Previous studies have demonstrated that cancer patients with higher levels of intratumor heterogeneity are associated with increased risk of postsurgical recurrence and worse treatment responses (Zhang *et al.* 2014, Reuben *et al.* 2017, Jia *et al.* 2018). Existing computational methods typically cluster cells or spots based on genomic profiles and pathological characteristics to facilitate regional description (Hu *et al.* 2021, Cable *et al.* 2022b). Alongside these approaches, others have explored different strategies, such as using graph deep learning to characterize tumor microenvironments from spatial protein profiles (Wu *et al.* 2022) and modeling intercellular communication in tissues using spatial graphs of cells (Fischer *et al.* 2023), which shared a similar goal of understanding tissue structure despite utilizing different technologies. However, for a comprehensive understanding of intra-sample heterogeneity, regions of interest are generally not those with a homogeneous constitution of a single cell type but rather those with highly interactive mixtures of different cell types. For instance, in tumor studies, regions with various infiltrating immune cells interacting with different tumor clones are of particular interest for understanding treatment responsiveness (Rosenberg *et al.* 1986, Salgado *et al.* 2015). To our knowledge, no existing methods can identify and characterize regions with high levels of interested-cell-type presence, highlighting an unmet need in this area.

Furthermore, commonly used platforms in spatially resolved transcriptomic sequencing, such as 10x Visium, Slide-seq (Rodriques *et al.* 2019), and high-definition spatial transcriptomics (HDST) (Vickovic *et al.* 2019), present additional challenges concerning cell type annotation and differential signal detection. These platforms often lack single-cell resolution or have limited coverage of sequenced genes, complicating the accurate inference of cellular identity and differential signal detection at the spot level. To address these challenges, several computational methods have been proposed to infer cellular composition by leveraging annotated single-cell RNA sequencing (scRNA-seq) data as a reference (Cable *et al.* 2022b, Ma and Zhou 2022). However, this information has not yet been systematically incorporated into cell type-specific differential gene detection, limiting our understanding of intratumor heterogeneity. More comprehensive methods that can effectively leverage cell compositions and enable more accurate differential gene expression analysis in spatial transcriptomics data are needed.

To address the aforementioned challenges, we introduce RegionalST, an efficient computational pipeline for identifying regions of interest (ROIs) and performing cross-region cell type-specific differential expression analysis. Unlike existing methods that typically divide samples into regions with homogeneous cell types, RegionalST identifies ROIs with high levels of cell type mixtures. This is achieved by using a weighted entropy algorithm that allows the specification of cell type importance and selects ROIs containing high enrichment of cell types with high biological relevance. Our proposed algorithm takes cellular compositions into consideration and has been shown to achieve higher accuracy of detecting true cell type-specific differential signals while maintaining fast computational speed. By incorporating both ROI identification and cell type-specific differential analysis into a single pipeline, RegionalST provides a one-stop solution for visualizing and analyzing diverse spatial transcriptomics data, enabling accurate and flexible exploration of tissue heterogeneity.

## 2 Materials and methods

**Figure 1.**
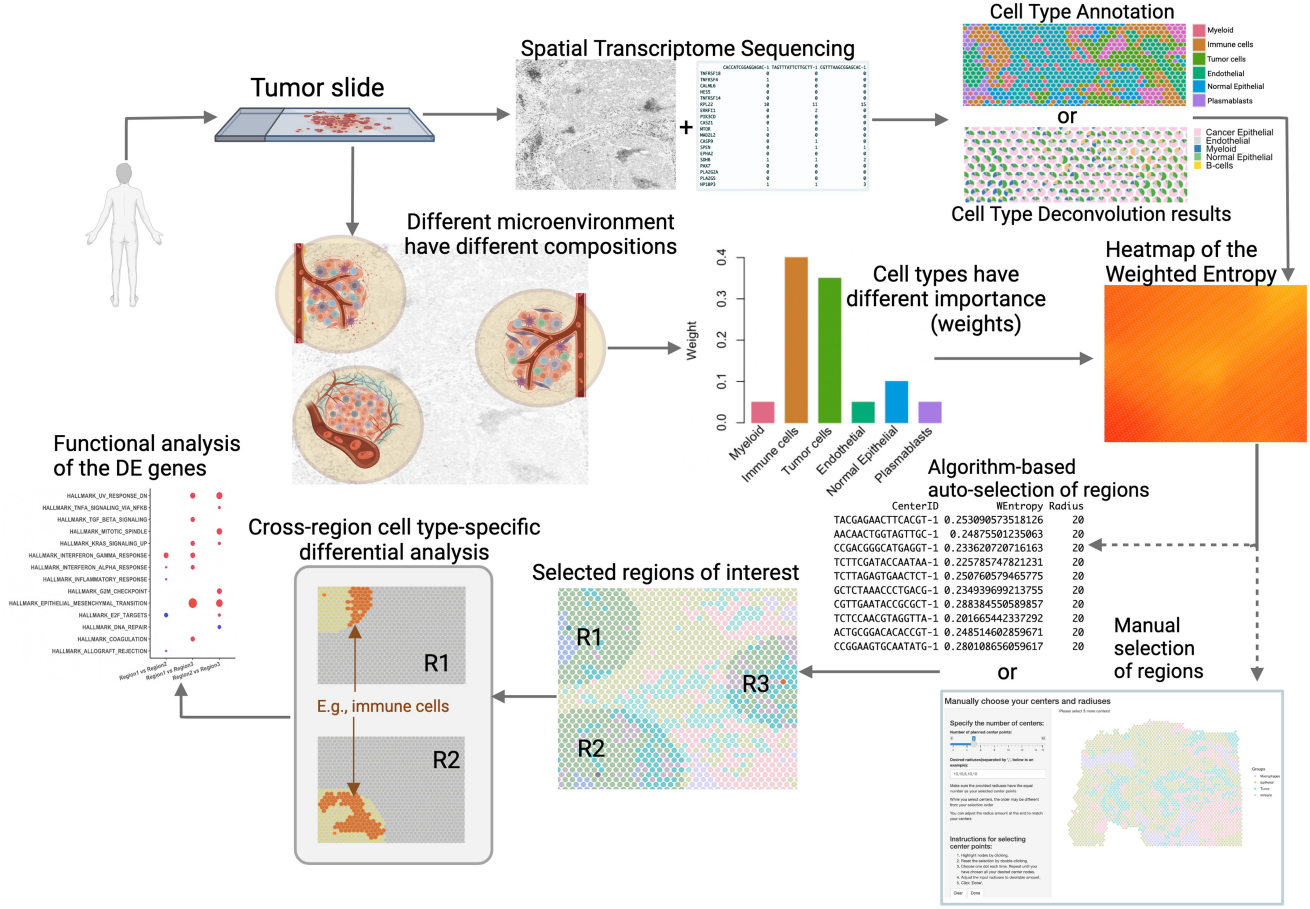
Schematic overview of a RegionalST workflow. Give the spatial transcriptomics data from a sample slide, the first step is to use biological markers to annotate cell types or to deconvolve the cell type proportion distribution using existing methods. Then the regional cell type interaction activity is quantified by entropy or weighted entropy. Regions of interest are selected by an algorithm-based method or manual selection. Cross-regional cell type-specific differential analysis and associated functional analysis is performed to provide insights on regional differences.

RegionalST consists of three steps for analyzing spatial transcriptomics data (Fig. 1). First, a cell-type mixture level heatmap is generated using entropy or weighted entropy, which requires cell type annotation or deconvolution results. Weighted entropy is then calculated for each spot at a fixed radius, giving greater weights to biologically important cell types (such as cancer epithelial or immune cells) and lower weights to other cell types (such as fibroblasts). The importance of each cell type is pre-specified and can be modified to emphasize different cell types according to research questions. The second step involves selecting ROIs based on the cell-type mixture level heatmap. The algorithm selects regions with the highest values of weighted entropy while maintaining a sufficient distance between regions to provide an adequate regional coverage. RegionalST provides an automatic algorithm for ROI selection, or users can manually select regions using an interactive shiny interface. Lastly, a cell type-specific differential expression analysis algorithm is implemented to detect cross-regional differential signals.

### 2.1 RegionalST analytical framework

The input of RegionalST requires two components: the spatial transcriptomics data and the spot-level cell type information (either cell type composition proportions or cell type annotations). Specifically, we denote the spatial transcriptomics gene expression profile by a G × S matrix W with rows as genes and columns as spots, and the associated spot location information is denoted by $\left\{(x_{s},y_{s}),s\in (1,2,\ldots ,S)\right\}$. For platforms where each spot consists of multiple cells, we can use existing methods like conditional autoregressive-based deconvolution (CARD) (Ma and Zhou 2022), robust cell type decomposition (RCTD) (Cable *et al.* 2022b), or cell2location (Kleshchevnikov *et al.* 2022) to obtain the spot-level cell type compositions, denoted by an S×K matrix H with rows as spots and columns as cell types. A detailed description of how we did estimation of cell composition is presented in Supplementary Section S1. In cases where the platform provides single cell resolution data or the deconvolution is inapplicable due to the lack of a matched single cell dataset, the spot-level cell type annotation is either obtained directly or by using the clustering and marker-based annotation approach such as BayesSpace (Zhao *et al.* 2021) and SpaGCN (Hu *et al.* 2021), denoted by a length *S* vector $M=\{m_{s},s=1,\ldots ,S\}$.

The first step of RegionalST is to construct candidate ROIs. For each spot *s*, the ROI with given center *s* and radius $r_{s}$ is defined as the set of spots satisfying

$$\begin{array}{c} \text{ROI}(s)=\{t:{\left[{\left(x_{t}\;-\;x_{s}\right)}^{2}+{\left(y_{t}\;-\;y_{s}\right)}^{2}\right]}^{1/2}\leq r_{s}\}, \end{array}$$

i.e. a set of spots that fall within the circle centered at *s* and with a radius of $r_{s}$ constitutes ROI. Given the cell type proportion matrix H, for spot *s*, we quantify the level of cell type mixture weighted entropy by

(1) $$\begin{array}{c} E(s,r_{s})=-\sum_{k=1}^{K}w_{k}p_{k}^{s}\;\text{log}(p_{k}^{s}), \end{array}$$

where $p_{k}^{s}=\sum_{t\in \mathrm{ROI}(s)}h_{tk}/\sum_{k^{\prime }}\left(\sum_{t^{\prime }\in \mathrm{ROI}(s)}h_{t^{\prime }k^{\prime }}\right)$, and $\left\{w_{k},k=1,\ldots ,K\right\}$ is the pre-specified cell type importance weight with $\sum_{k=1}^{K}w_{k}=1$. If the weights are unspecified, we use the unweighted entropy by default with $w_{k}=1/K,k\in \{1,\ldots ,K\}$. When the cell type annotation vector M is available, we compute the regional cell type proportion by $p_{k}^{s}=\sum_{t\in \mathrm{ROI}(s)}I\{m_{t}=k\}/\sum_{k^{\prime }}(\sum_{t^{\prime }\in \mathrm{ROI}(s)}I\{m_{t^{\prime }}=k^{\prime }\})$ where I{·} is the indicator that equals 1 when $m_{t}=k$ is true and 0 otherwise(details of illustration of weighted entropy can be found at).

Next, we select the top ROIs based on their weighted entropy values. We design the following automatic algorithm to identify the top *Q* ROIs that have the highest values of cell type mixtures and at the same time maintain a minimum distance among the centers to avoid regions overlapping. Although Algorithm 1 performs the weighted entropy calculation over all the spots for each radius option, which can be time consuming for large datasets, we find that taking a random subset of the spots as candidate centers achieves a similar performance as iterating through all spots. In our implementation, we randomly select about one tenth of the spots as candidate centers. It should be noted that our radius selection does not consider differential analysis results, and thus would not purposely identify regions with more differentially expressed genes (DEGs). As the algorithm is purely based on the weighted entropy calculations and does not consider other biological factors, users might want to customize the ROI selection. To meet such needs, we also develop our interactive Shiny application for ROI selection. Our ROI selection approach implicitly makes two assumptions about the association between cell type interaction and their proportions in each region. First, the co-existence of more cell types usually indicates a higher chance of cellular interactions across those cell types, regardless of their spatial distribution within the region. And second, more interaction occurs as that mixture approaches the user-specified cell type weight matrix or uniform distribution if weight matrix not provided.


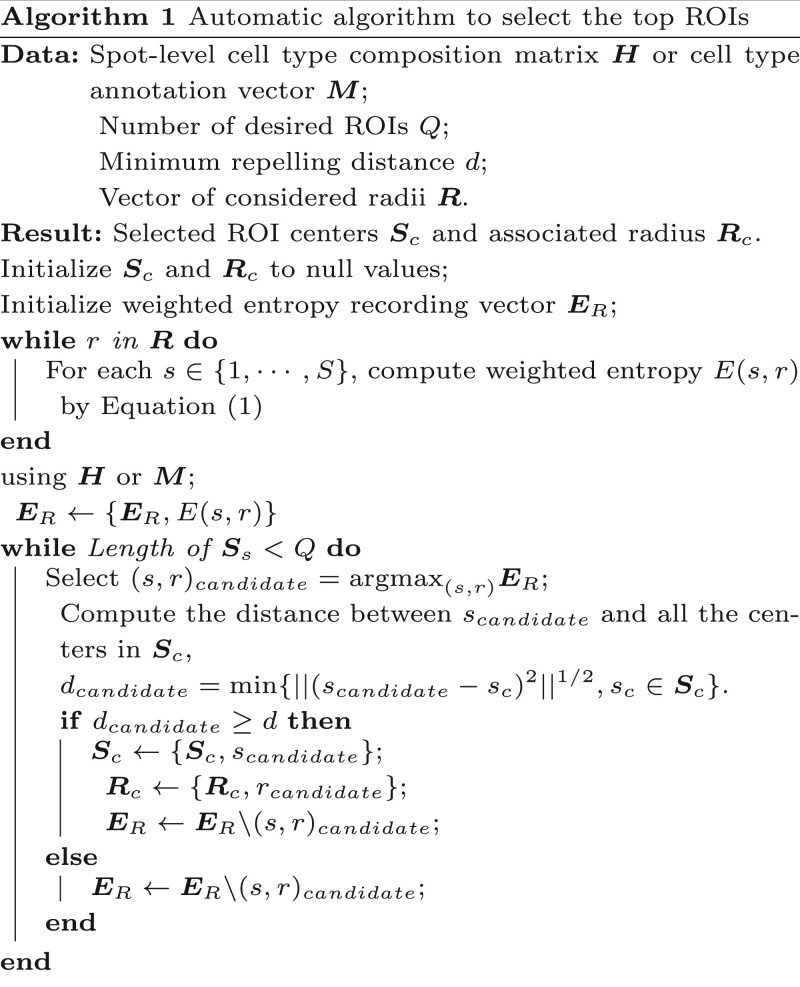


After selecting the top ROIs, RegionalST performs cross-regional differential analysis by comparing the ROIs in a pairwise way. Assume the spatial transcriptomic gene expression profile of the $q_{1}$th ROIs as $W_{q_{1}}$. Let us consider a simple comparison between two ROIs, $W_{q_{1}}$ and $W_{q_{2}}$. Assume the spatial transcriptomics data have been properly normalized and thus can be modeled using Gaussian distribution. If the data have single-cell resolution or have been annotated to major cell types based on the marker information, the spots with the same cell type from $W_{q_{1}}$ and $W_{q_{2}}$ can be directly examined and compared using a Wilcoxon test (Hao *et al.* 2021) or MAST (Finak *et al.* 2015). When each spot consists of multiple cell types and the cell type compositions are available to be adjusted for in the differential analysis, we denote the associated cell type proportion matrix for the two ROIs as $H_{q_{1}}$ and $H_{q_{2}}$. $H_{q}=\{H_{q_{1}},H_{q_{2}}\}$. We consider a linear model framework with

$$\begin{array}{c} E(W_{i})=\sum_{k=1}^{K}E(X_{ik})H_{ik},\;i\in \{q_{1},q_{2}\}, \end{array}$$

where $X_{q_{1}}$ and $X_{q_{2}}$ are the mean cell type-specific gene expression at the two regions, and *K* is the total number of cell types. To test whether cell type *k* has differential expressions in the two ROIs, $X_{i}$ can be modeled by incorporating the ROI information as a covariate $X_{ik}=\alpha_{k}+\beta_{k}Z_{i}$ where $Z_{i}=0$ if $i=q_{1}$ and $Z_{i}=1$ if $i=q_{2}$. Together, the model of $W_{q}=\{W_{q_{1}},W_{q_{2}}\}$ can be written as

$$\begin{array}{c} w_{i}=\sum_{k=1}^{K}\left(h_{ik}\alpha_{k}+h_{ik}\beta_{k}Z_{i}\right),\;\;i=1,\ldots ,G, \end{array}$$

where $w_{i}$ and $h_{i}$ are the *i*th row of matrices $W_{q}$ and $H_{q}$. This model coincides with the cell type-specific differential analysis considered in problems of bulk RNA-seq (Li *et al.* 2019). The difference is that the W here is the spot-level transcriptome profile. Note that the spatial information is used by H and thus the similarity between neighboring regions is considered through the modeling of H. Following existing works with similar purpose, we do not explicitly model the location in the differential analysis (Cable *et al.* 2022a). We use an F test with null hypothesis $H_{0}:\beta_{k}=0$ to statistically examine the significance of comparing the two regions $q_{1}$ and $q_{2}$ in cell type *k*. The Benjamini–Hochberg procedure is applied to control the false discovery rate (FDR) less than 5%.

### 2.2 Interactive Shiny app for ROI selection

In addition to the automatic ROI selection algorithm, we also provide an interactive shiny app to facilitate manual selection of the ROI centers and radii. The application is implemented using the R Shiny package, which features a straight-forward sample visualization panel, a graphical user interface (GUI) accompanied by detailed tutorials with example data. Users can begin by selecting the desired number of ROIs and tentative radii. ROI centers are then chosen interactively by clicking on spots within the interactive sample figure. The click-recording function is supported by the “clickOpts()” tool from the R Shiny app. Based on the locations of the selected centers, users can change the radiuses to desired values in order to minimize overlapping regions. After all the ROIs are selected, the centers and the radiuses are the output from the interactive app for further downstream analysis.

## 3 Results

### 3.1 Simulation

We first evaluated the performance of RegionalST using simulated spatial transcriptomics datasets, which were generated based on a real scRNA-seq dataset with Dirichlet-distributed cellular compositions and fully defined cell type-specific differential genes in the single-cell references (Zheng *et al.* 2017) (Details refer to Supplementary Section S2). The simulated dataset consisted of four cell types, CD4 T helper cells, B cells, CD14 monocytes, and CD56 NK cells, with each spot containing 20 individual cells. When combining the single cell to spot level gene expression profiles, we randomly selected 500 genes in the first cell type and 300 genes in the second cell type as DEGs. To generate single-cell gene expression data of the DEGs, we imposed fold changes on the cells from the case group.

**Figure 2.**
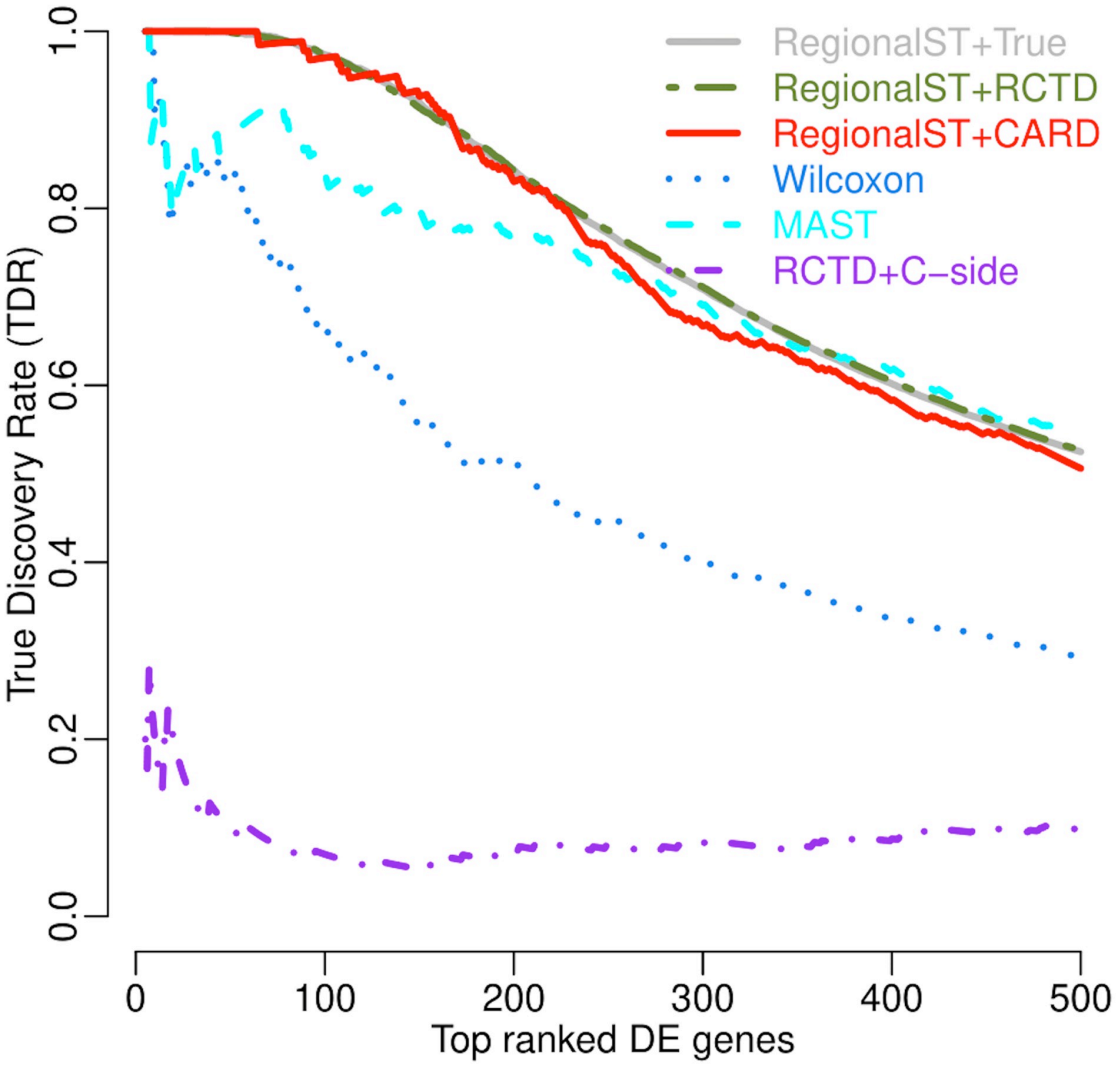
Simulation results comparison with existing methods. Evaluated differential analysis: RegionalST+TRUE (RegionalST with true cell type proportions); RegionalST+RCTD (RegionalST with cell type proportions estimated by RCTD); RegionalST+CARD (RegionalST with cell type proportions estimated by CARD); Wilcoxon and MAST, single cell differential analysis method by Seurat package; RCTD+C-side (C-side with cell type proportions estimated by RCTD).

We compared the proposed method, RegionalST, with other existing methods, C-Side (Cable *et al.* 2022a), Wilcoxon (Hao *et al.* 2021), and MAST (Finak *et al.* 2015). Wilcoxon and MAST treat each spot as a single cell, ignoring the cell type mixture in the data. In addition, we considered different cell type deconvolution methods and thus evaluated RegionalST plus true proportions, RegionalST plus cell type proportions estimated by RCTD, and RegionalST plus CARD. Our simulation results showed that incorporating the cellular compositions could effectively improve differential signal detection (Fig. 2 for settings with 10% of genes as DE genes and Supplementary Fig. S11 in Supplementary material for settings with 6% DE genes). Specifically, RegionalST achieved a consistently higher true discovery rate (TDR) in the top 500 ranked DE genes. Moreover, RegionalST performed robustly with different deconvolution methods in both settings, achieving better or comparable results as MAST and better performance than Wilcoxon or RCTD plus C-Side.

### 3.2 Real data results

#### 3.2.1 Application to breast cancer datasets

**Figure 3.**
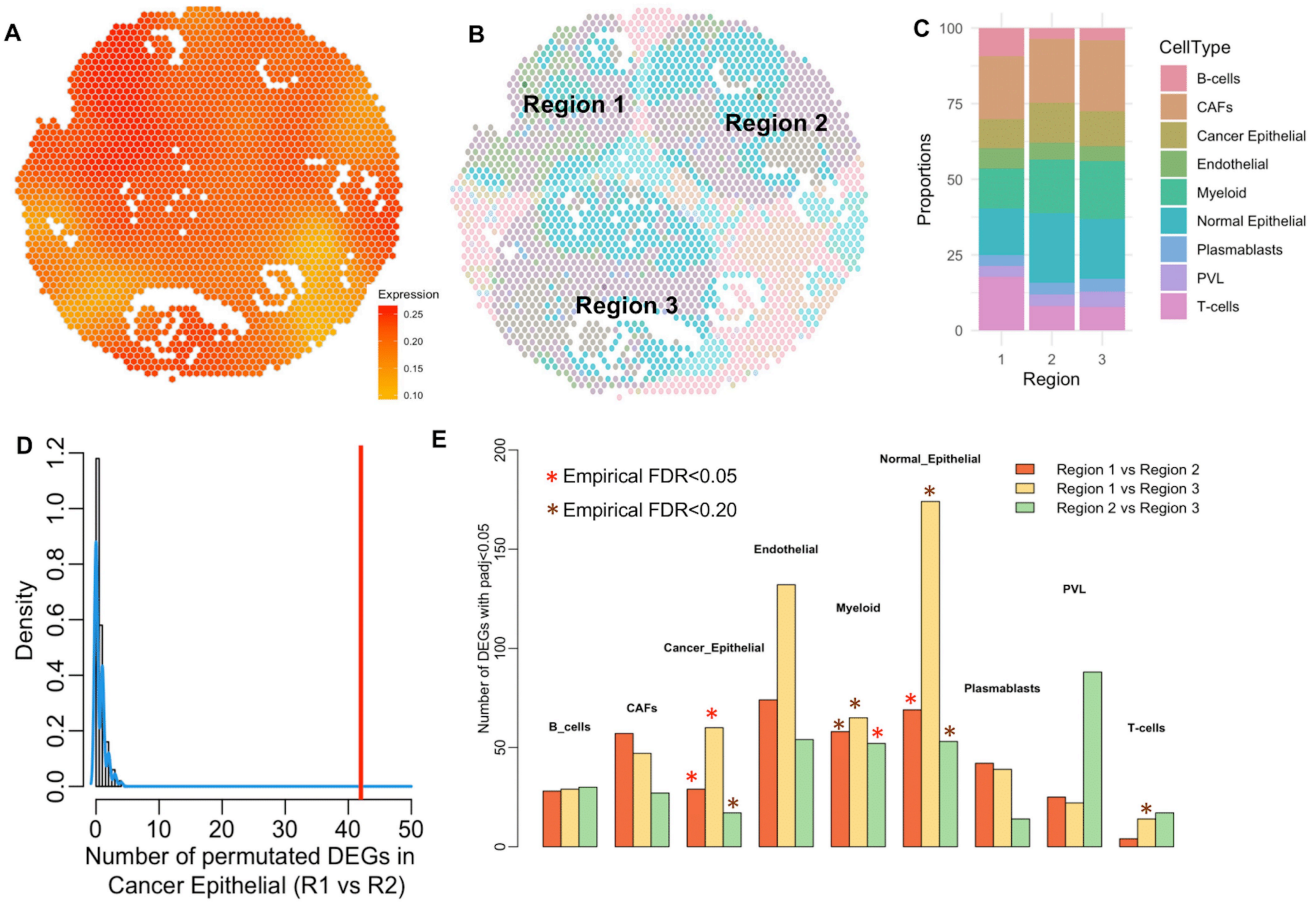
Results of applying RegionalST to two breast cancer spatial transcriptomics datasets and an ovarian cancer dataset. (A) Weighted entropy of the tumor slide based on the spatial transcriptomics data. (B) ROIs in the first breast cancer data using a radius size of 15. The detailed cell type labels associated with (B) are available in Supplementary Fig. S1. (C) The proportional distribution of the cell types in the three ROIs. (D) Number of permutated DEGs in cancer epithelial by comparing Regions 1 and 2 in 100 permutation experiments (gray bars, distribution fitted by blue line) and the number of DEGs detected by RegionalST (red line). (E) Barplot of the DEG numbers with adjusted *P* value smaller than 0.05 in different cells types and different comparisons. The stars indicate the result of empirical FDRs. Small empirical FDRs are labeled and suggest that the observed DEG number is much larger than the permutation distribution of the DEG number in the comparison.

Next, we applied RegionalST to two breast cancer spatial transcriptomics datasets obtained by 10X Visium. The pathology plot and the gene marker heatmap of the first dataset (Supplementary Figs S1A and S2) showed that the epithelial cells (tumor and nontumor) were presented around the several duct areas, while the immune cell types (T cells and B cells) distribute mainly around the top left area and near the slide border. We applied CARD (Ma and Zhou 2022), a computational method that borrows information across nearby spots, to estimate cell type composition of each spot (Supplementary Fig. S1B, with implementation details in the Supplementary Section S1). To obtain ROIs, we used the cellular composition result from CARD and specified a higher weight for cancer epithelial, B-cells, and T cells. The cell type weighted entropy heatmap showed more cell interaction enrichment mostly in the top area and the duct area near the bottom border (Fig. 3A). Consistently, our automatic regional selection algorithm chose three regions at the top left, top right, and the bottom areas of the slide as ROIs (Fig. 3B). The ROI selection stayed the same if we used a different deconvolution method, RCTD. We observed that both CARD and RCTD achieved highly accurate (and similar) cell type deconvolution results.

Additional analysis of these three ROIs suggested important differences in cell type distributions among the regions. Region 1 has more immune cells and fewer normal epithelial cells compared to the other two regions (Fig. 3C). We performed cross-regional cell type-specific DE analysis. To examine the validity of the detected DEGs, we designed a permutation procedure by permuting the spots in the two comparing regions and regenerating the number of DEGs. Note that each spot contains multiple cells that may come from different cell types. If the number of DEGs identified from the permuted data is much smaller than the number of DEGs from nonpermuted data, it implies a small false discovery rate. Reassuringly, we observed such a pattern in the permutation results (see Fig. 3D for cancer epithelial cells comparing Region 1 versus 2 as an example). We summarized the validity obtained by the permutation test as empirical FDRs and included the information in the barplot of the cross-regional DEGs (Fig. 3E). In this application, the results from cancer epithelial, myeloid, normal epithelial cells and one comparison in T cells have better statistical validity compared to the other cell type-specific comparisons.

**Figure 4.**
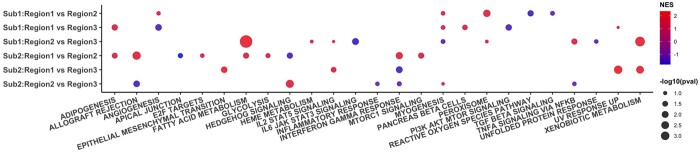
Hallmark analysis result of the cross-regional DEG analysis for the two subjects in cancer epithelial cells

We also observed that the cell type abundance can impact the validity of the cross-regional DEG detection. In the second breast tumor dataset, the majority of the spots consists of cancer epithelial cells and the rarity of other cell types greatly impacts the cell type-specific DEG analysis (Supplementary Fig. S4C). Cell types that are less abundant tend to have less information in the data and thus the DEG in these cell types is harder to be detected accurately. Due to the impact of uneven cell type proportions, the comparison between two datasets based on the identified DEGs need to be extra careful. As a result, we only obtained low empirical FDRs for the cross-regional analysis in cancer epithelial cells. Comparing the cross-regional results from the two breast cancer patients in cancer epithelial, we identified several hallmark pathways enriched in both subjects, including fatty acid metabolism, xenobiotic metabolism, and TNFA (tumor necrosis factor) signaling via NF-κB (Nuclear factor-κB) (Fig. 4, details in the Supplementary Section S6). Interestingly, there are substantial variations in the highlighted pathways between the two subjects. For example, interferon gamma response is only reported in the cross-regional analysis for the second subject but not the first, while angiogenesis and myogenesis are mostly observed in the first patient. Interferon gamma plays context-dependent dual tumor-suppressor and pro-tumorigenic roles in breast cancer (Tecalco-Cruz *et al.* 2021). The up- and down-regulation of interferon gamma response may be associated with intratumor heterogeneity in the first patient. In contrast, angiogenesis and myogenesis have been reported to promote tumor cell progression and dissemination (Longatto Filho *et al.* 2010). Clinical information indicates that the first breast cancer patient has a lower grade than the second patient (grade II versus III). The enrichment of these two pathways may be associated with the worse stage condition in the second patient.

#### 3.2.2 Application to ovarian cancer dataset

**Figure 5.**
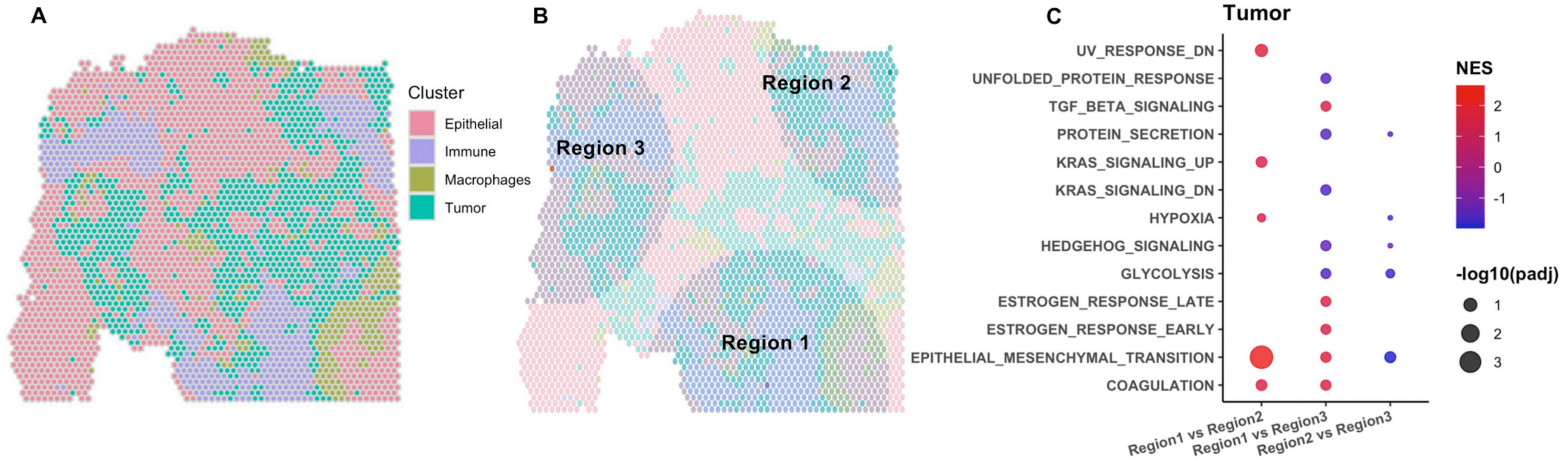
Results of analyzing the ovarian tumor sample. (A, B) The cell type annotation and ROIs of the ovarian tumor spatial transcriptomics dataset using a radius size of 20. (C) Hallmark enrichment of the cross-regional analysis in tumor cell.

An ideal scRNA-seq reference for cell type deconvolution may not always exist. We examined the proposed method in such a scenario with an ovarian cancer spatial transcriptomics dataset. We divided the spots into nine clusters using BayesSpace and examined known biological markers to annotate the clusters into four major cell types (Fig. 5A, with implementation details in the Supplementary Section S3). Three regions with a mixture of immune and tumor cells were observed, and these three regions were suggested by RegionalST as ROIs (Fig. 5B). When comparing the tumor cells in the three regions, we observed a significant enrichment in the epithelial mesenchymal transition (EMT) pathway in Region 1 compared to Region 2 (Fig. 5C). Previous findings have indicated that ovarian cancer is a highly metastatic disease, and the activation of EMT plays a key role in cancer invasion and metastasis (Vergara *et al.* 2010, Deng *et al.* 2016). Moreover, several other tumor metastasis-associated pathways have also been identified, including the KRAS (Kirsten rat sarcoma virus) signaling pathway (e.g. in tumor), coagulation (e.g. in tumor and immune cells), and interferon gamma response (e.g. in immune cells), when comparing Region 1 to the other regions (Supplementary Fig. S7). These findings suggest that the tumor clones in Region 1 could be more aggressive, presenting a higher risk of metastasis compared to the other clones in this tumor sample (Windbichler *et al.* 2000, Wang *et al.* 2005, Kim *et al.* 2020). Furthermore, RegionalST has identified a number of differentially expressed genes that play important roles in ovarian cancer progression, such as *COL1A1* (Li *et al.* 2020), *GNAS* (Tominaga *et al.* 2010), and *TP53* (Milner *et al.* 1993). Therefore, this cross-regional analysis helps explain the heterogeneous response of tumor treatment and offers opportunities to identify clone-specific drug targets.

## 4 Discussion

In summary, RegionalST is a new tool for identifying ROIs from spatial transcriptomics data, enabling cross-regional differential analysis. Unlike existing methods that cluster spatial spots based on their similarities, RegionalST identifies ROIs by a novel weighted entropy system that depicts the level of interested-cell-type presence, along with the flexibility to focus on regions enriched with key cell types of interest. Importantly, comparing with existing clustering methods that often prioritize clustering spots from the same cell type, RegionalST emphasizes regions with mixtures of multiple cell types and avoids cell type-dominated results (Supplementary Fig. S10). In addition, due to small cell numbers and/or inaccurate estimates of cell type composition, the model-based cell type-specific DE tests may have an inflated type I error. RegionalST uses a permutation procedure to evaluate empirical FDRs to identify such situations. The pipeline has been implemented as a user-friendly software that allows both manual and automatic selection of ROIs as well as cross-regional differential expression analysis with the incorporation of cell type compositions. Moreover, RegionalST can be readily extended to accommodate situations with multiple samples, facilitating the understanding of cross-sample and cross-subject heterogeneity.

For recent platforms, such as MerFISH and CosMX, that provide data at single-cell resolution, clustering and marker-based cell type annotation are sufficient to accurately depict the cell type abundance. However, when using common platforms such as Visium 10X and Slide-seq, we recommend applying deconvolution algorithms, such as CARD or RCTD, for more precise quantification of the cell type constitutions. Using both simulated and real datasets, we demonstrate the superior accuracy and interpretability of RegionalST compared with existing methods. We anticipate that RegionalST will prove useful for delineating the within-tumor heterogeneity and cross-regional microenvironment differences in diverse pathological environments.

To demonstrate the robustness of our chosen ROI radius sizes, we conducted additional simulation study and real data applications to evaluate the different choices of ROI radius size (details in Supplementary material Section S4). However, one limitation of the current framework is that we only consider centered ROIs. Future work of adopting arbitrary-shaped ROIs associated with the tissue structure will address this limitation and improve the ROI selection performance. In addition, although weighted entropy allows searching for regions that enriched with specific cell types, it should be noted that the method relies on prior knowledge of cell type significance provided by users. This input can be quite subjective and context dependent, leading to inconsistent findings by different users. Acknowledging this limitation is crucial for the appropriate interpretation of the weighted entropy and emphasizes the need for sensitivity analysis when using this approach in practice.

We also recognize that our current tool has not been specifically tailored for analyzing single-resolution datasets. Although we examined our method on single-resolution Xenium data (details in Supplementary Section S5 and Supplementary Fig. S12), how to better utilize the high-resolution data and provide best analytical results for analyzing single-cell-resolution data is an unsolved question. Another limitation is that our current ROI selection algorithm considers a series of pre-specified radii. Data-adaptive approaches can be developed in future works to automatically select the most appropriate ROI-specific radius. While the current framework can be applied to multi-subject datasets, many factors, such as within-subject and cross-subject heterogeneity, are not systematically evaluated; they should be considered in future extensions.

## Supplementary Material

btae186_Supplementary_Data

## Data Availability

All spatial transcriptomics data were downloaded from the 10X website (https://www.10xgenomics.com/resources/datasets). The PBMC single cell dataset is downloaded from http://support.10xgenomics.com/single-cell/datasets.
