# Supplementary material for: Regional analysis to delineate intrasample heterogeneity with RegionalST
Source: Bioinformatics. 2024 Apr 5;40(4):btae186. doi: 10.1093/bioinformatics/btae186 (PMC11026142; doi:10.1093/bioinformatics/btae186)
Supplement: btae186_Supplementary_Data [file btae186_supplementary_data.pdf]

Supplementary materisl for "Regional analysis to delineate intrasample heterogeneity with RegionalST"

Yue Lyu[1,2], Chong Wu[1], Wei Sun[3,4,5], Ziyi Li[1,*]

[1]Department of Biostatistics,
The University of Texas MD Anderson Cancer Center, Houston, TX 77030, USA.
[2]Department of Biostatistics and Date Science,
The University of Texas Health Science Center at Houston, Houston, TX 77030, USA.
[3]Biostatistics Program, Public Health Science Division,
Fred Hutchinson Cancer Center, Seattle, WA 98109, USA.
[4]Department of Biostatistics,
University of North Carolina at Chapel Hill, Chapel Hill, NC 27516, USA.
[5]Department of Biostatistics,
University of Washington, Seattle, WA 98195, USA.

[*] Correspondance: zli16@mdanderson.org.

S1. Estimation of cell type compositions

Many existing methods are available to estimate cell type compositions from the spatial transcriptomics data, including CARD [1], RCTD [2], cell2location [3], and SpatialDWLS [4]. In our experiments, we use CARD to deconvolve the spatial transcriptomics data from the two breast cancer patients. CARD uses a conditional autoregression-based model by combining cell type-specific expression data from scRNA-seq and the spatial information from the spatial transcriptomics. They assume that the spot-level cell type compositions have higher correlations with the neighboring regions and thus the model borrows information using an autoregressive structure. The default CARD function has been applied to solve for cell type compositions and the visualization is performed using the functions "CAR.visualize.pie" and "CARD.visualize.prop". When drawing the pie plot, we modify the "CAR.visualize.pie" function to accept smaller pie radius to avoid overlapping pie charts.

S2. Details of simulation setting

We design a simulation study to evaluate the performance of RegionalST in identifying cell type-specific differential genes when each spot of spatial transcriptomics data consists of multiple cells. We generate the simulation data based on a real scRNA-seq dataset [5] and use the data from four cell types, CD4 T helper cells, B cells, CD14 monocytes, and CD56 NK cells. To mimic the situation where the distributions of different cells types varies across spatial locations, we randomly generate the cell type compositions of each

spot using the following rule:

$$\boldsymbol{h}_s \begin{cases} \sim \text{Dirichlet}(5,3,1,1), & \text{with probability } 0.2 \\ \sim \text{Dirichlet}(2.5,2.5,2.5,2.5), & \text{with probability } 0.6 \\ \sim \text{Dirichlet}(3,5,1,1), & \text{with probability } 0.2 \end{cases} .$$

In other words, the four cell types evenly distributed in about 60% of the spots while the first or second cell type is the dominant cell type in the rest of the spots. When combining the single cell to spot level gene expression profiles, we manually select 500 genes in the first cell type and 300 genes in the second cell type as the differentially expressed genes. For genes from the second region, we impose a fold change that follows a uniform distribution from 1.5 to 2.5, while the single cells to construct the first region are randomly drawn from the real dataset without imposing any fold change. With these procedures, we generated 200 spots per region and each spot consists of 20 single cells. We present the true discovery rate versus the gene ranking for RegionalST, Wilcoxon [6], and MAST [7]. Both Wilcoxon and MAST are applied by treating the spots as single cells, and we use the default parameter setting. The results are averaged over 100 Monte Carlo simulations.

S3. Analysis of spatial transcriptomics datasets

After the data were downloaded from the 10X website (see "Data Availability" section), we read in the data and perform preprocessing with BayesSpace. For the two breast cancer datasets, we apply CARD and obtain the cell type-specific compositions (see "Estimation of cell type compositions" section). For ovarian cancer data, we cluster the spots using the "spatialCluster" function in BayesSpace and label the cell type identity with marker information (EPCAM for epithelial; EPCAM, MUC6, MMP7 for tumor cells; CD14 and CSF1R for macrophages; CCR7 for dendritic cells; CD19, CD79A, CD79B, and PTPRC for immune cells) following a previous study [8].

S4. Choice of radius sizes, repelling distance threshold and number of desired ROIs

We conducted additional simulations and real data analysis using different choices of radius sizes. In the simulation study, we considered regions with number of spots ranging from 150 to 400 (corresponding to radius from 8 to 13) and evaluated the accuracy of RegionalST versus the underlying true DEs (generated in the simulation procedure). Considering the total number of spots in the breast cancer Visium dataset is 2518, 150 is about 6% and 400 is 16% of the spots. These are reasonable sizes and not very big. We find that the increase of region sizes indeed result in better differential signal detection, which is understandable that larger regions provide larger spot numbers(Fig S8). From 150 to 400, the true discovery rate stays higher than existing method Wilcoxon and RCTD+C-Side, while the performance is comparable between MAST and RegionalST 100/200 and better in RegionalST 250 and above.

We also examined different radius sizes in real data analysis with the breast cancer Visium data (Fig S9). We compared the results using radius being 10 ($\sim$ 250 spots), 11 ($\sim$ 300 spots), 12 ($\sim$ 350 spots), and 13 ($\sim$ 400 spots). The sets of identified differentially expressed genes are largely overlapped when a difference of 50 spots exists. Even with a larger difference by radius being 10 and 13, the overlapped DE genes are still around 30 percent. It should be noted that we don't expect zero changes in DEGs when we increase the

radius size, because larger ROIs include more spots that were not considered in the analysis with smaller ROIs and thus introduce new DE signals.

The selection of minimum repelling distance depends on several factors, such as the technology and the choice of ROI radius. A smaller repelling distance (e.g., 5 - 10) is usually preferred for technology such as 10x ST as only a few hundreds of spots are measured. But for 10x Visium or Xenium, a larger repelling distance (e.g., 10 - 15) is better to ensure sufficient consideration of the whole slide. The choice of ROI radius also play an important role in selecting repelling distance. We recommend a repelling distance that is similar or larger than the ROI radius to avoid overlapping ROIs.

The natural restriction of the ROI number is the total measured spots and the repelling distance. Fewer total measured spots and larger repelling distance leads to less available ROI selections. Except these factors, users can select as many ROIs as needed, if the selected ROIs are interpretable and biologically meaningful.

S5. Robustness to choice of deconvolution method

We conducted additional simulations by including different deconvolution methods (RegionalST+RCTD, RegionalST+CARD) versus using true cell type proportion (RegionalST+True). , We considered the settings where 10% of the genes are DE genes (Fig 2) and 6% of the genes are DE genes (Fig S11). We find that RegionalST performs robustly with different deconvolution methods in both settings, achieving better or comparable results as MAST and better performance than Wilcoxon or RCTD+C-Side.

We also explored to apply our proposed method on single-resolution data. We downloaded a Xenium dataset of human skin melanoma sample slide (https://www.10xgenomics.com/resources/datasets/human-skin-preview-data-xenium-human-skin-gene-expression-panel-add-on-1-standard). Based on the annotation of cell type markers (Immune: CD3D, CD3E, MS4A1, CD79A; MoMac: LYZ, CD68, CD14; Endothelial: VWF, PECAM1; Melanoma: MLANA, PMEL, MITF, DCT; Fibroblast: COL1A1, COL3A1), we annotated the cell types labels at the single cell resolution (Fig S12, panel A and B). RegionalST identified five multiple-cell type mixed regions (Fig S12, panel C) and we performed differential analysis as well as downstream pathway analysis (Fig S12, panel D). We found that Region 2 and 3 have less G2M checkpoint activated comparing to Region 3, while Region 2 is also less activated in allograft rejection comparing to Region 4. Nonetheless, we acknowledge that our current tool has not been specifically tailored for analyzing single-resolution datasets. Comparing to Visium and ST platforms, the single-cell-resolution technology generally results in much larger datasets and smaller number of genes sequenced. As a result, how to better utilize the high-resolution data and provide best analytical results for analyzing single-cell-resolution data is an interesting question and will be address in our future works.

S6. Functional enrichment analysis

To facilitate functional interpretation of the cross-regional differential analysis, we output the full lists of all the testing results and apply gene set enrichment analysis (GSEA) using the fgsea R package [9]. GSEA examines the ranking of the genes from a given pathway in the differential analysis and identifies the pathways that are enriched at the top or bottom of the ranked list. We provide our differential analysis results ranked by the effect size (the effect change in β with respect to the magnitude in α, a measurement similar to log fold change). In our implementation, we consider three in-built databases for GSEA analysis, including the Hallmark [10], KEGG [11], and Reactome [12]. Hallmark pathway is generally recommended when cancer-related cells are under consideration, while the other databases are suitable for more general analysis.

S7. Illustration of weighted entropy

To better understand when the entropy will be big/small, we have provided a visual illustration of the relationship between entropy, cell type proportion, and assigned cell type weights (Fig S15). This figure shows the exact value of cell type mixture weighted entropy in five different scenarios, each differing by cell type compositions and the assigned weights. In the yellow panel, a region with only a few cell types where cancer and immune cells are given higher weights yields the highest entropy value (0.31). The blue panel represents a region with a few cell types where cancer and immune cells are assigned lower weights, leading to the lowest entropy value (0.03). The pink panel shows a region with multiple cell types, all assigned unequal weights, resulting in a slightly lower entropy (0.28). The purple panel shows a region with few cell types, all assigned unequal weights, resulting in a lower entropy (0.17). The green panel depicts a scenario with multiple cell types with equal weights resulting in a slightly lower entropy (0.26). We observed that when the cell type proportion has consistent patterns with the assigned weights, the cell type mixture achieves the highest value. In scenarios where some cell types (like cancer and immune cells) are prevalent and given higher weights, the entropy value increases. This aligns with the concept of weighted entropy, where the contribution of each cell type to the entropy calculation is scaled by its assigned weight, reflecting its biological importance. Therefore, regions with a high proportion of biologically significant cell types, which are also heavily weighted, will naturally have higher entropy values.

S8. Single-cell resolution real data analysis

We also explored to apply our proposed method on single-resolution data. We downloaded a Xenium dataset of human skin melanoma sample slide (https://www.10xgenomics.com/resources/datasets/human-skin-preview-data-xenium-human-skin-gene-expression-panel-add-on-1-standard). Based on the annotation of cell type markers (Immune: CD3D, CD3E, MS4A1, CD79A; MoMac: LYZ, CD68, CD14; Endothelial: VWF, PECAM1; Melanoma: MLANA, PMEL, MITF, DCT; Fibroblast: COL1A1, COL3A1), we annotated the cell types labels at the single cell resolution (Figure below panel A and B; Figure also available in Supplementary material Figure S12). RegionalST identified five multiple-cell type mixed regions (panel C) and we performed differential analysis as well as downstream pathway analysis (panel D). We found that Region 2 and 3 have less G2M checkpoint activated comparing to Region 3, while Region 2 is also less activated in allograft rejection comparing to Region 4. Nonetheless, we acknowledge that our current tool has not been specifically tailored for analyzing single-resolution datasets. Comparing to Visium and ST platforms, the single-cell-resolution technology generally results in much larger datasets and smaller number of genes sequenced. As a result, how to better utilize the high-resolution data and provide best analytical results for analyzing single-cell-resolution data is an interesting question and will be address in our future works.

References

[1] Ying Ma and Xiang Zhou. Spatially informed cell-type deconvolution for spatial transcriptomics. Nature biotechnology, 40(9):1349–1359, 2022.

[2] Dylan M Cable, Evan Murray, Luli S Zou, Aleksandrina Goeva, Evan Z Macosko, Fei Chen, and Rafael A Irizarry. Robust decomposition of cell type mixtures in spatial transcriptomics. Nature Biotechnology, 40(4):517–526, 2022.

[3] Vitalii Kleshchevnikov, Artem Shmatko, Emma Dann, Alexander Aivazidis, Hamish W King, Tong Li, Rasa Elmentaite, Artem Lomakin, Veronika Kedlian, Adam Gayoso, et al. Cell2location maps fine-grained cell types in spatial transcriptomics. Nature biotechnology, 40(5):661–671, 2022.

[4] Rui Dong and Guo-Cheng Yuan. Spatialdwls: accurate deconvolution of spatial transcriptomic data. Genome biology, 22(1):145, 2021.

[5] Grace XY Zheng, Jessica M Terry, Phillip Belgrader, Paul Ryvkin, Zachary W Bent, Ryan Wilson, Solongo B Ziraldo, Tobias D Wheeler, Geoff P McDermott, Junjie Zhu, et al. Massively parallel digital transcriptional profiling of single cells. Nature communications, 8(1):14049, 2017.

[6] Yuhan Hao, Stephanie Hao, Erica Andersen-Nissen, William M. Mauck III, Shiwei Zheng, Andrew Butler, Maddie J. Lee, Aaron J. Wilk, Charlotte Darby, Michael Zagar, Paul Hoffman, Marlon Stoeckius, Efthymia Papalexi, Eleni P. Mimitou, Jaison Jain, Avi Srivastava, Tim Stuart, Lamar B. Fleming, Bertrand Yeung, Angela J. Rogers, Juliana M. McElrath, Catherine A. Blish, Raphael Gottardo, Peter Smibert, and Rahul Satija. Integrated analysis of multimodal single-cell data. Cell, 2021.

[7] Greg Finak, Andrew McDavid, Masanao Yajima, Jingyuan Deng, Vivian Gersuk, Alex K Shalek, Chloe K Slichter, Hannah W Miller, M Juliana McElrath, Martin Prlic, et al. Mast: a flexible statistical framework for assessing transcriptional changes and characterizing heterogeneity in single-cell rna sequencing data. Genome biology, 16(1):1–13, 2015.

[8] Benjamin Izar, Itay Tirosh, Elizabeth H Stover, Isaac Wakiro, Michael S Cuoco, Idan Alter, Christopher Rodman, Rachel Leeson, Mei-Ju Su, Parin Shah, et al. A single-cell landscape of high-grade serous ovarian cancer. Nature medicine, 26(8):1271–1279, 2020.

[9] Gennady Korotkevich, Vladimir Sukhov, Nikolay Budin, Boris Shpak, Maxim N Artyomov, and Alexey Sergushichev. Fast gene set enrichment analysis. BioRxiv, page 060012, 2016.

[10] Arthur Liberzon, Chet Birger, Helga Thorvaldsdóttir, Mahmoud Ghandi, Jill P Mesirov, and Pablo Tamayo. The molecular signatures database hallmark gene set collection. Cell systems, 1(6):417–425, 2015.

[11] Minoru Kanehisa and Susumu Goto. Kegg: kyoto encyclopedia of genes and genomes. Nucleic acids research, 28(1):27–30, 2000.

[12] Bijay Jassal, Lisa Matthews, Guilherme Viteri, Chuqiao Gong, Pascual Lorente, Antonio Fabregat, Konstantinos Sidiropoulos, Justin Cook, Marc Gillespie, Robin Haw, et al. The reactome pathway knowledgebase. Nucleic acids research, 48(D1):D498–D503, 2020.

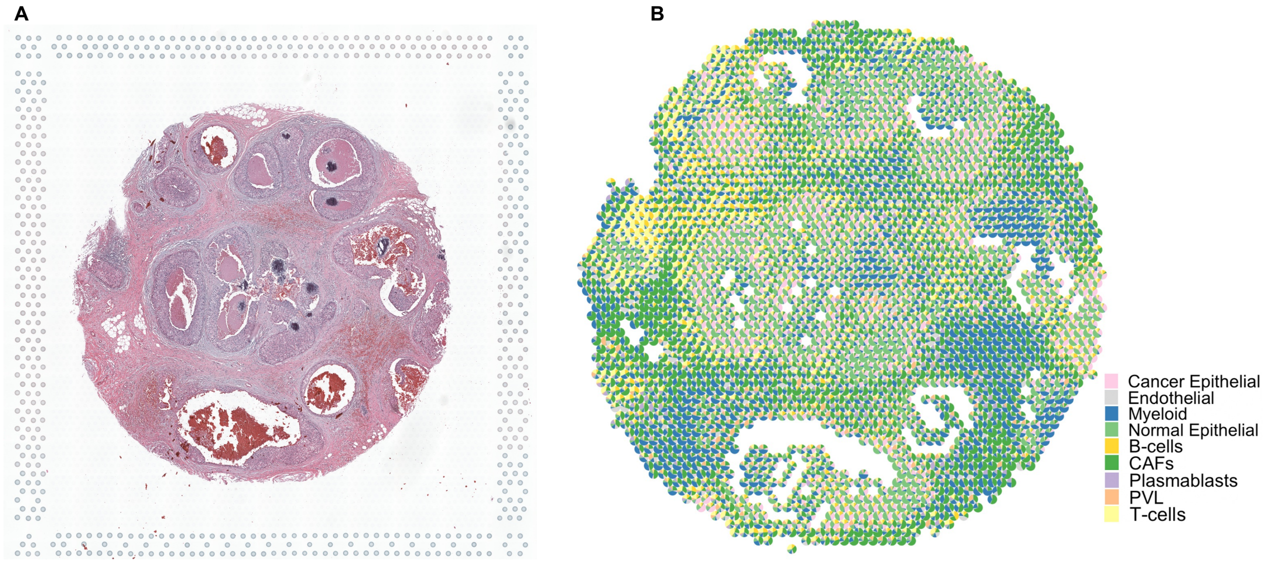

Figure S1: **Pathology and deconvolution results of the FFPE human breast ductal carcinoma sample.** (A) pathology of the tumor slide from the first breast cancer patient. (B) distribution of the cell type proportions estimated by conditional autoregressive-based deconvolution (CARD).

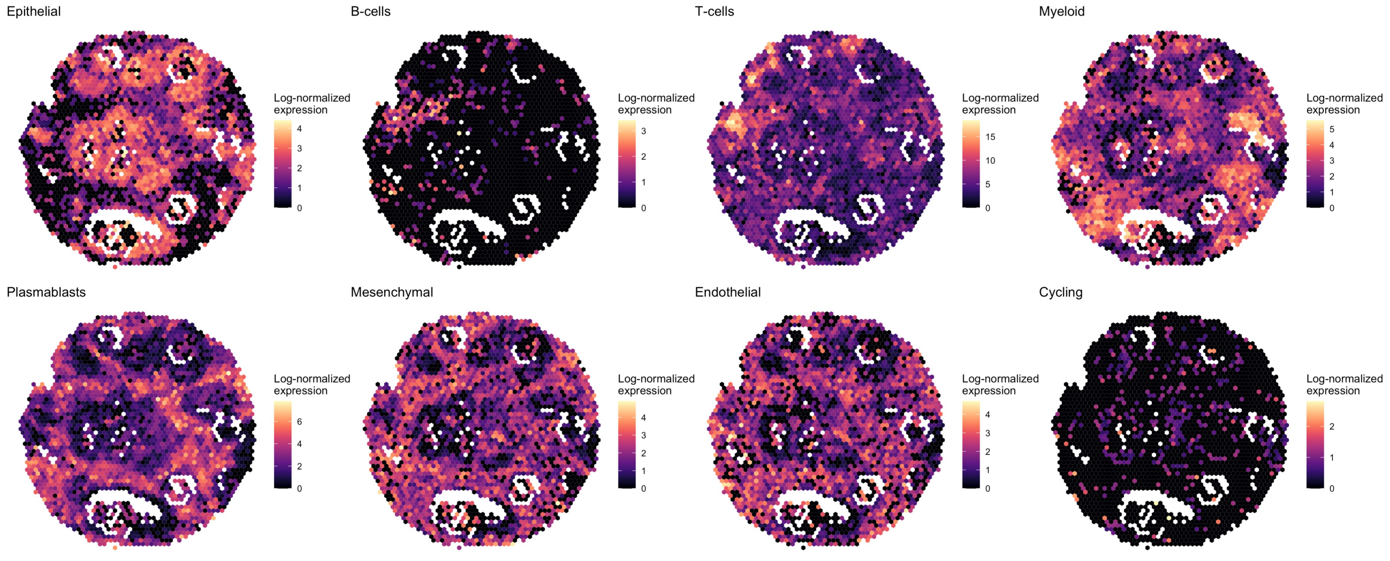

Figure S2: Visualization of the log-normalized expression level of the cell type-specific markers for eight non-tumor cell types using the FFPE human breast ductal carcinoma data.

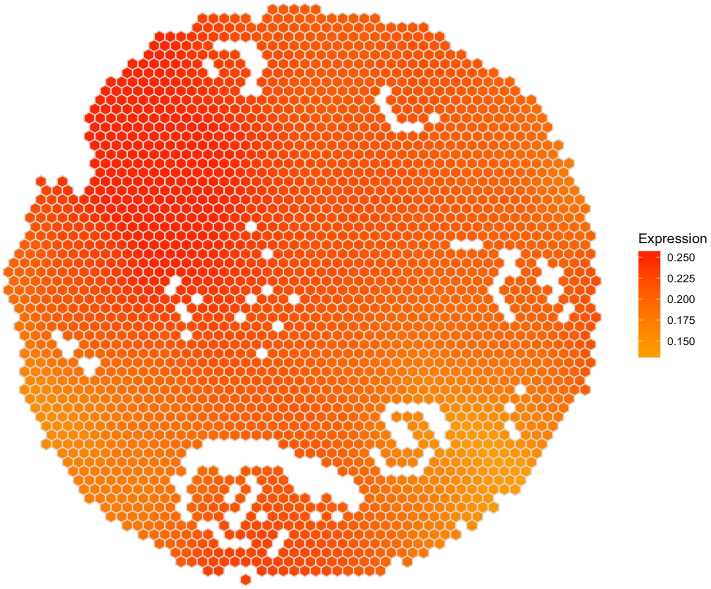

Figure S3: Weighted entropy of the FFPE breast ductal carcinoma spatial transcriptomics data using radius 10.

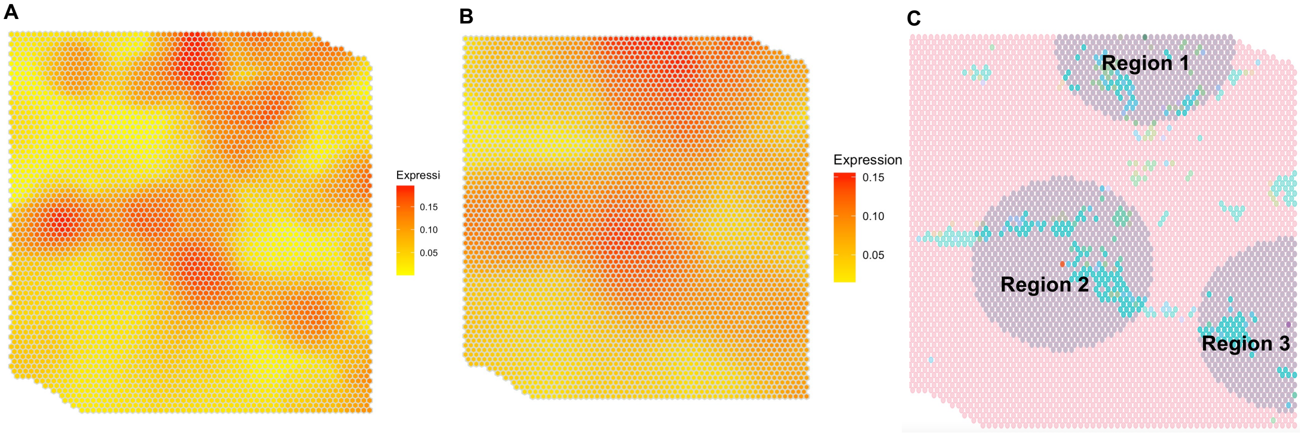

Figure S4: Weighted entropy of the frozen fresh breast cancer data using radius 5 (panel A) and 10 (B). (C) ROIs selected by RegionalST from the frozen fresh breast cancer data.

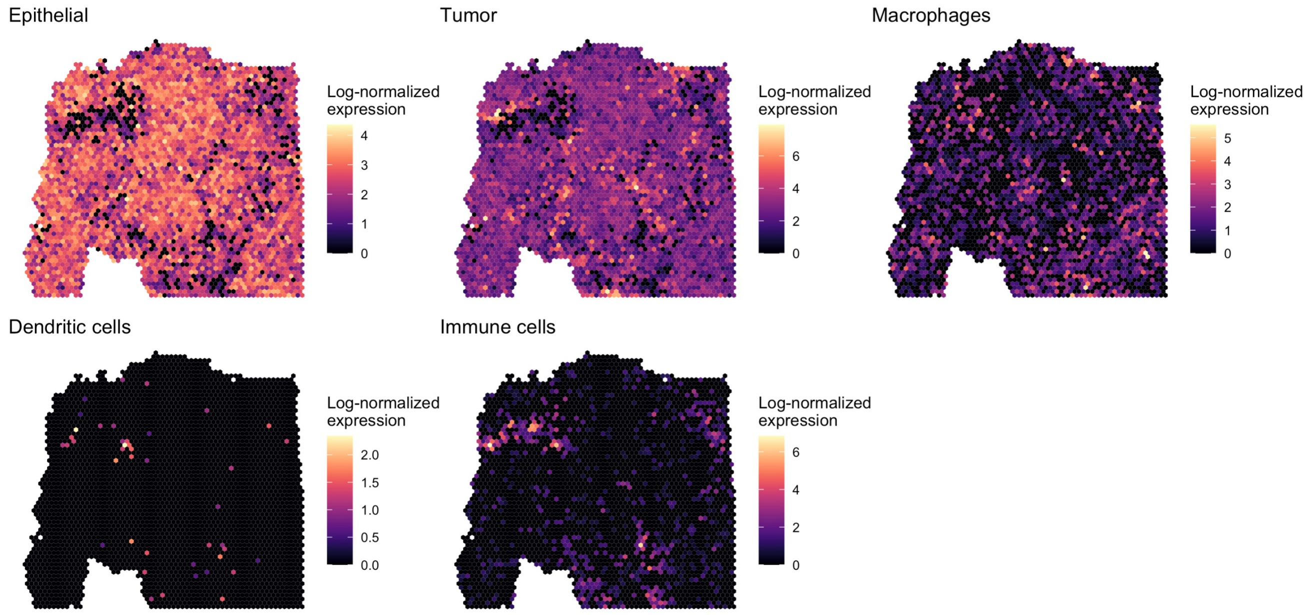

Figure S5: Visualization of the log-normalized expression level of the cell type-specific markers for the five major cell types using the ovarian cancer data.

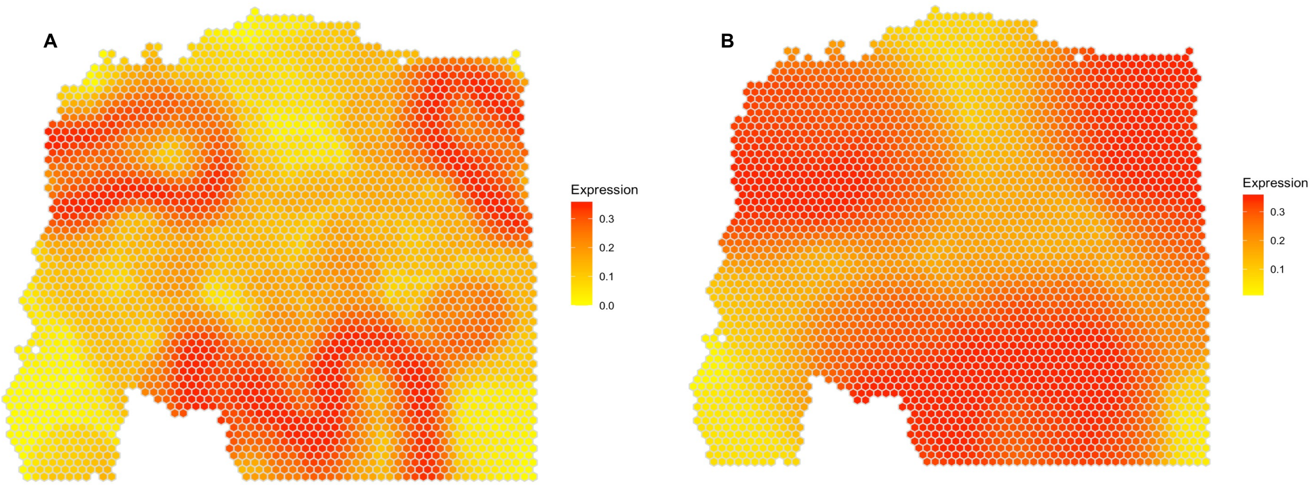

Figure S6: Weighted entropy of the ovarian cancer data using radius 5 (panel A) and 10 (panel B).

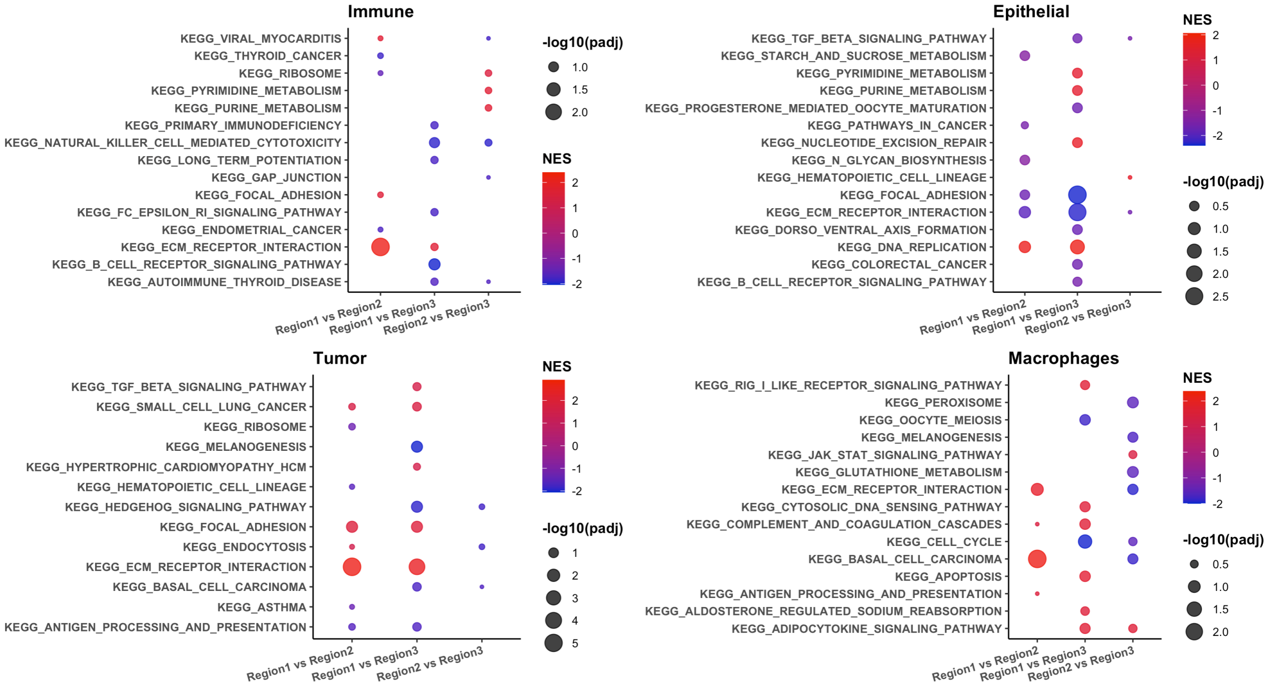

Figure S7: Gene Set Enrichment Analysis (GSEA) results of the cross regional cell type-specific differentially expressed genes for the immune, epithelial, tumor, and macrophage cells.

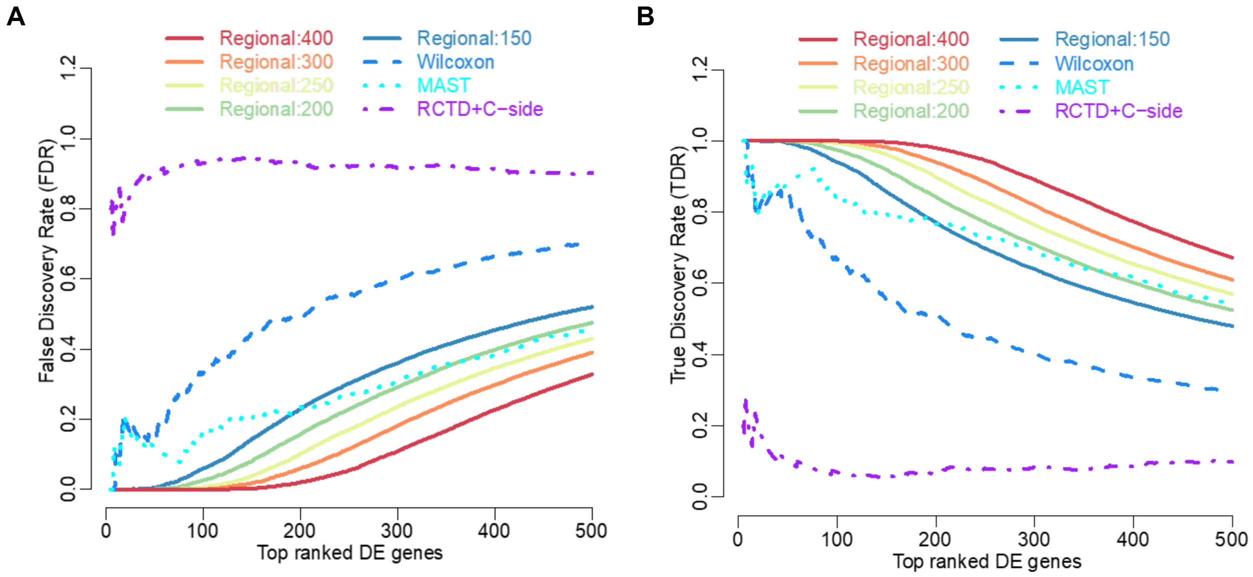

Figure S8: **Simulation results of evaluating the different choices of ROI radius sizes.** Left panel: false discovery rate by the top ranked DE genes; right panel: true discovery rate top ranked DE genes.

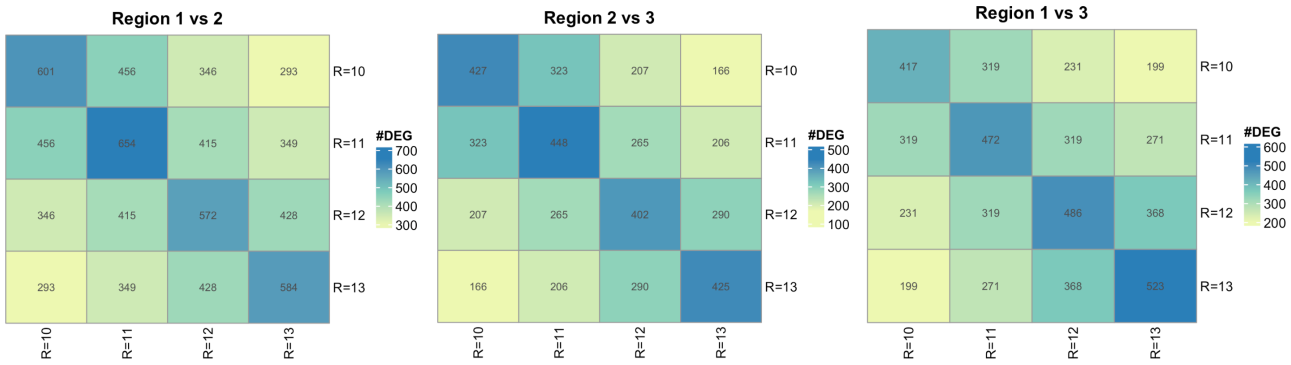

Figure S9: Heatmap for the number of overlapped DE genes using different radius sizes in breast cancer data analysis.

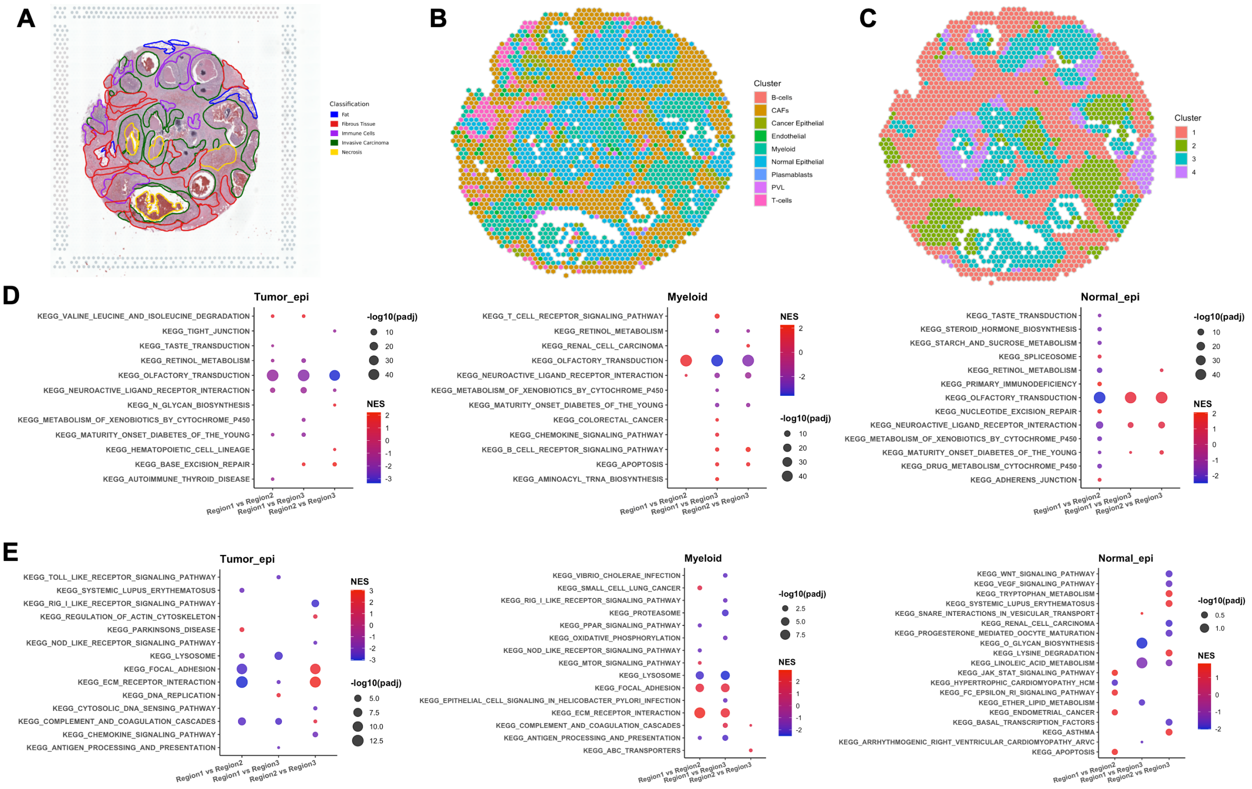

Figure S10: **Results of applying BayesSpace on the breast cancer Visium data.** A, pathologist annotated slide; B, cell type results based on the CARD deconvolution estimation; C, clustering results using BayesSpace; D, KEGG pathway enrichment results using the BayesSpace cluster 1, 2, and 3; E, KEGG pathway enrichment results using RegionalST.

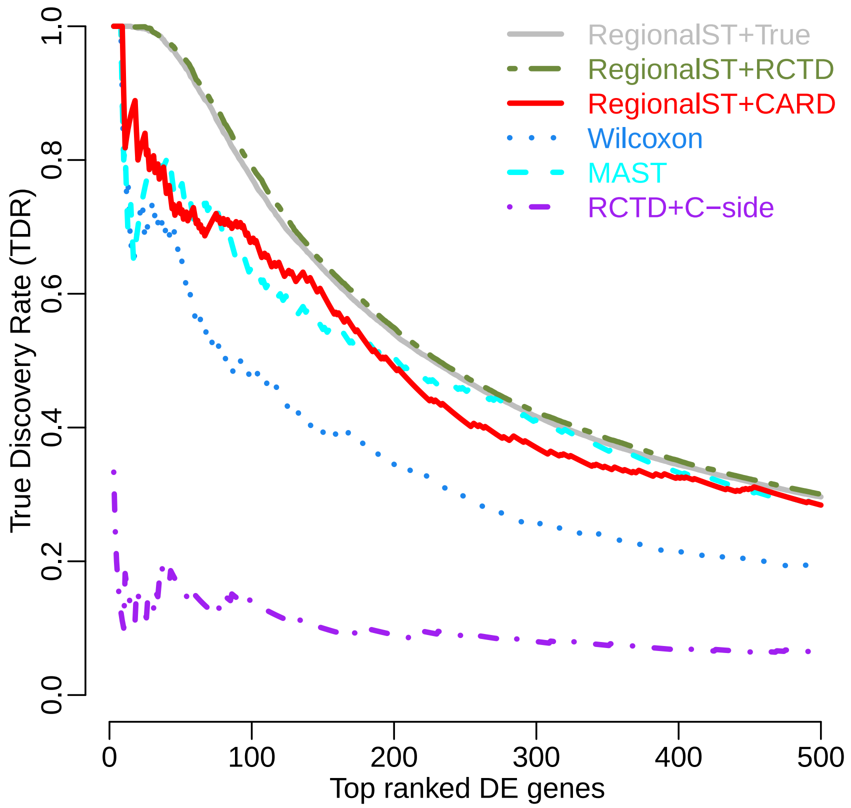

Figure S11: Simulation results with 6% of genes as DEG. Evaluated differential analysis method: RegionalST+TRUE (RegionalST with true cell type proportions); RegionalST+RCTD (RegionalST with cell type proportions estimated by RCTD); RegionalST+CARD (RegionalST with cell type proportions estimated by CARD); Wilcoxon and MAST, single cell differential analysis method by Seurat package; RCTD+C-side (C-side with cell type proportions estimated by RCTD).

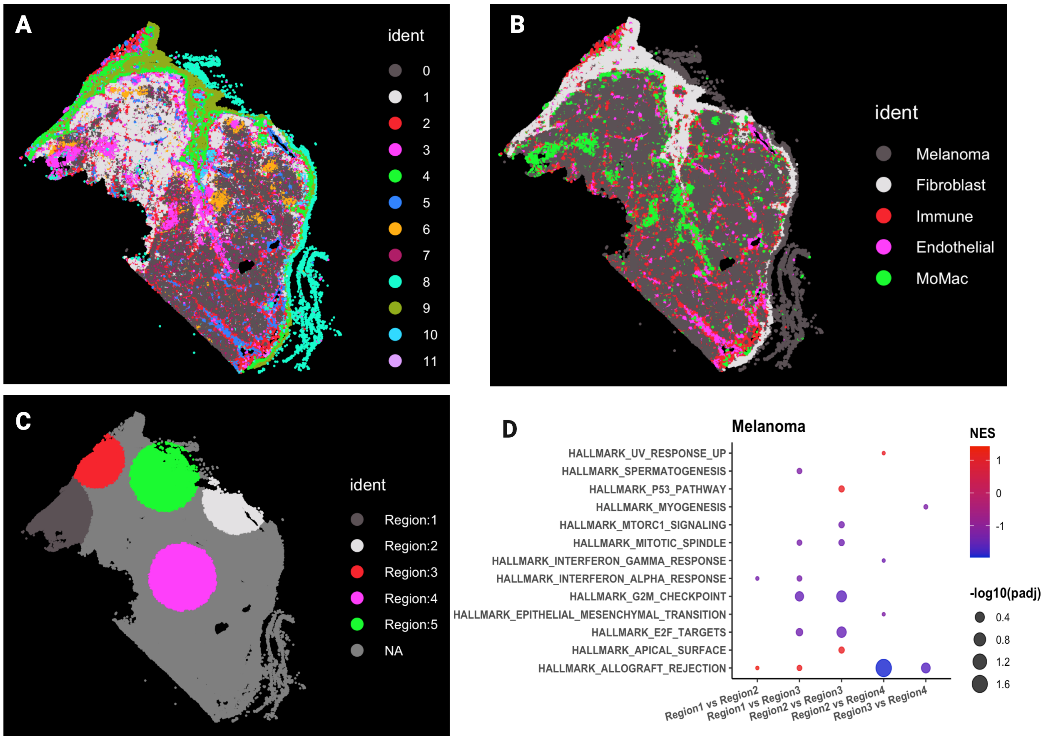

Figure S12: **Analysis results using the human skin melanoma Xenium dataset.** A, clustering results by Seurat; B, cell type label assignment based on marker genes; C, ROIs selected by RegionalST; D, hallmark enrichment analysis results based cross-cell type differential genes.

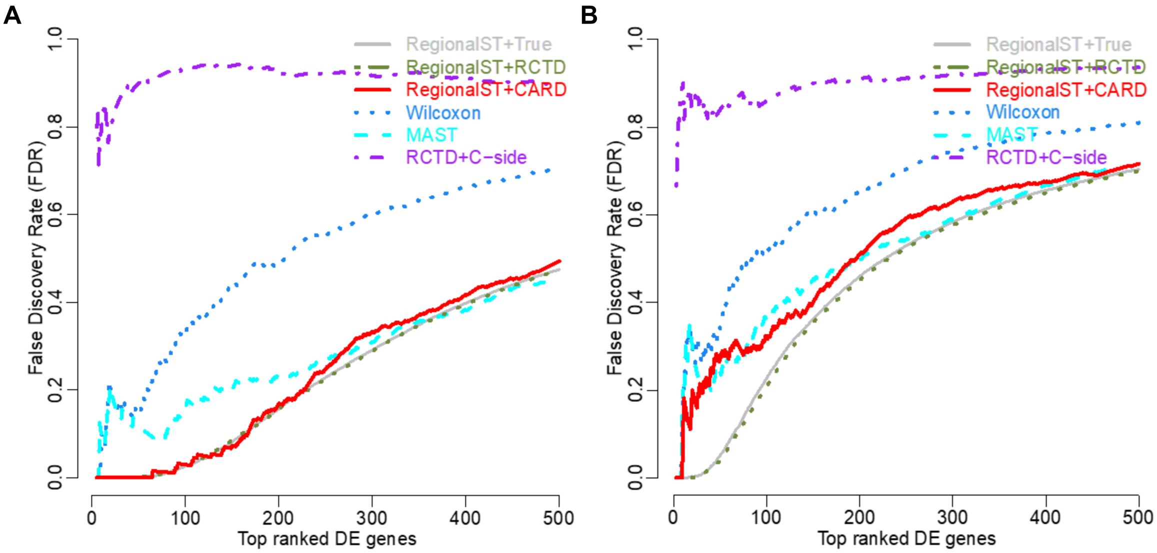

Figure S13: False discovery rate of evaluated methods when 10% of the genes are underlying DEG (A) and 6% are DEG (B).

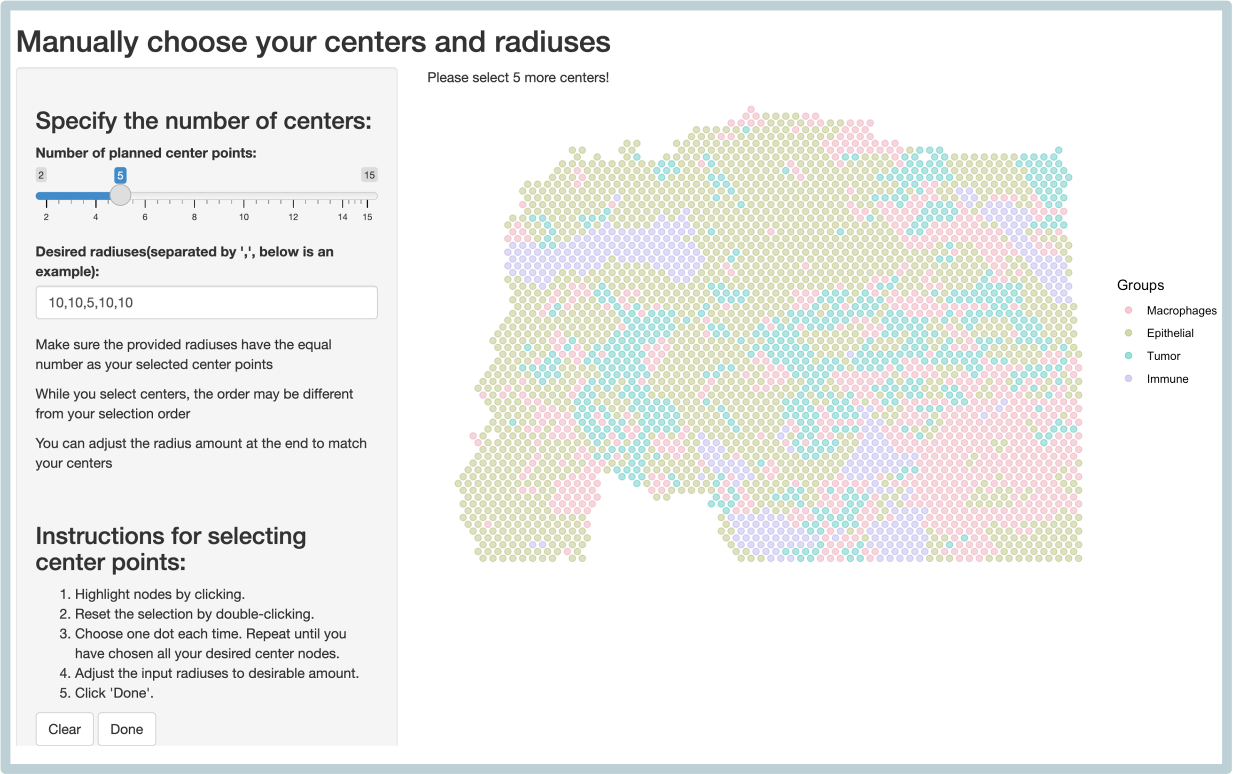

Figure S14: Screenshoot of the Shiny Application.

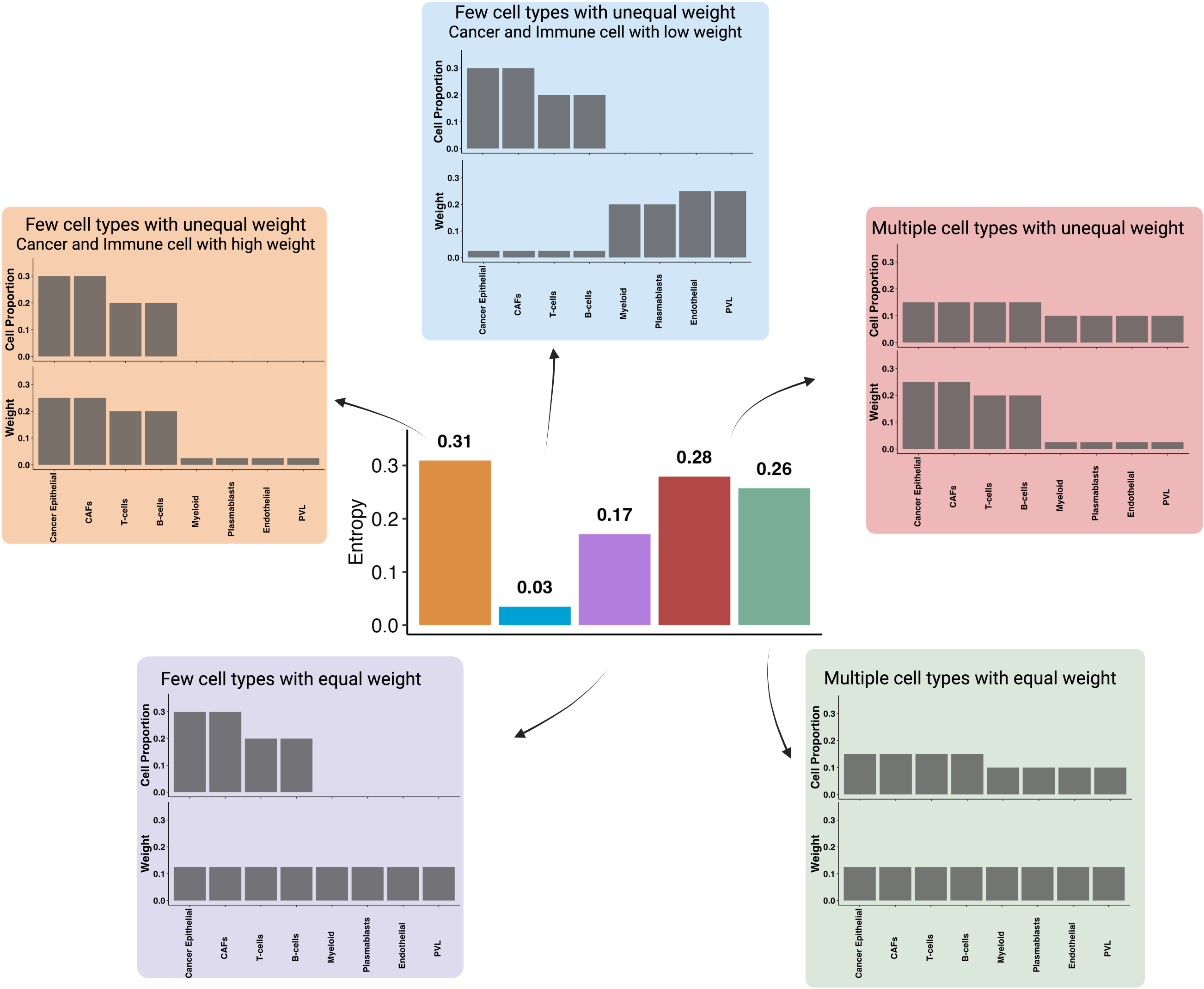

Figure S15: Illustration of the relationship between entropy, cell type proportion, and assigned cell type weights.
